# Cargo selection in endoplasmic reticulum–to–Golgi transport and relevant diseases

**DOI:** 10.1172/JCI163838

**Published:** 2023-01-03

**Authors:** Vi T. Tang, David Ginsburg

**Affiliations:** 1 Department of Molecular and Integrative Physiology,; 2 Life Sciences Institute,; 3 Department of Internal Medicine,; 4 Department of Human Genetics,; 5 Department of Pediatrics and Communicable Diseases, and; 6 Howard Hughes Medical Institute, University of Michigan, Ann Arbor, Michigan, USA.

## Abstract

Most proteins destined for the extracellular space or various intracellular compartments must traverse the intracellular secretory pathway. The first step is the recruitment and transport of cargoes from the endoplasmic reticulum (ER) lumen to the Golgi apparatus by coat protein complex II (COPII), consisting of five core proteins. Additional ER transmembrane proteins that aid cargo recruitment are referred to as cargo receptors. Gene duplication events have resulted in multiple COPII paralogs present in the mammalian genome. Here, we review the functions of each COPII protein, human disorders associated with each paralog, and evidence for functional conservation between paralogs. We also provide a summary of current knowledge regarding two prototypical cargo receptors in mammals, LMAN1 and SURF4, and their roles in human health and disease.

## Introduction

Cells secrete proteins into the extracellular environment for multiple purposes, including intercellular communication, defense, and modulation of the external environment. In eukaryotes, the majority of secreted proteins must transit through a series of membrane-bound organelles before arriving at the plasma membrane for release into the extracellular environment (1–3). This pathway is also shared by many proteins destined for the cell surface and various intracellular organelles, such as endosomes and lysosomes (2, 3). Proteins enter the intracellular secretory pathway at the endoplasmic reticulum (ER), where they are cotranslationally embedded into the ER membrane or deposited into the ER lumen (4, 5). Properly folded proteins are then transported from the ER to the Golgi apparatus for further processing and sorting before reaching their final extracellular or intracellular destinations (6, 7). A few proteins have been shown to exit the cell via an ER/Golgi–independent process referred to as unconventional protein secretion (8–11). This Review will focus on the early phase of conventional protein secretion, in which proteins are transported from the ER to the Golgi in a process mediated by coat protein complex II (COPII). Genetic disorders associated with deficiency in protein components of COPII will also be described.

## ER to Golgi transport by COPII

### COPII composition.

**Figure 1:**
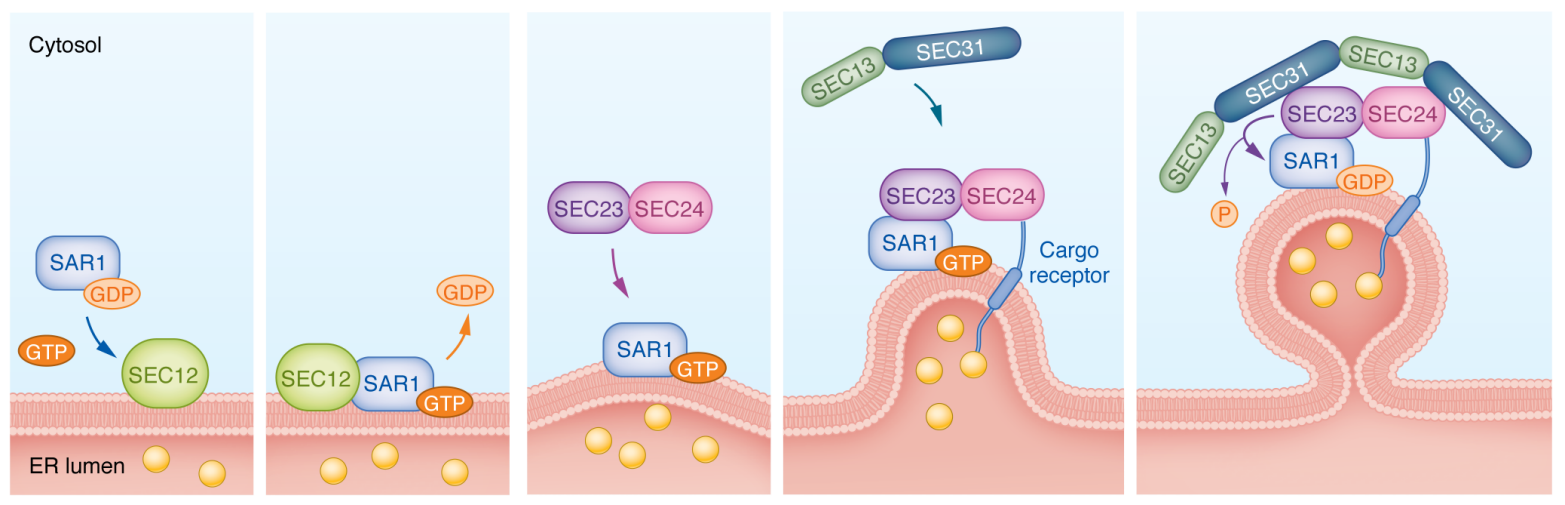
COPII coat assembly on the ER membrane. SEC12 recruits GDP-bound SAR1 to ER exit sites (ERESs) and acts as a guanine nucleotide exchange factor for SAR1. GTP-bound SAR1 inserts its hydrophobic N-terminus into the ER membrane and recruits SEC23-SEC24 heterodimers to the ER exit site via direct interaction with SEC23. SEC24 mediates cargo recruitment via direct physical interaction with transmembrane proteins through their cytoplasmic tails or with soluble cargoes via cargo receptors. SEC23 also functions as the GTPase-activating protein for SAR1 and stimulates SAR1 GTP hydrolysis. Finally, SEC13-SEC31 heterotetramers are recruited as the outer coat to complete the coat assembly process.

The components of the COPII coat were first identified through a genetic screen by Randy Schekman and colleagues in 1980 for *Saccharomyces cerevisiae* mutants that were defective in protein secretion, designated “sec” mutants (12). The core COPII components in *S*. *cerevisiae* are Sar1p, Sec23p, Sec24p, Sec13p, and Sec31p (13). The corresponding vertebrate proteins are referred to as SAR1, SEC23, SEC24, SEC13, and SEC31. SAR1 is a small GTPase that recruits other coat proteins to the ER membrane. SEC23 and SEC24 form an inner coat complex that is proximal to the ER membrane. SEC13 and SEC31 form the outer coat complex (Figure 1).

### Coat assembly.

COPII coat assembly begins in the membrane of the smooth ER (lacking ribosomes) where the localization of SEC12 defines the ER exit site (ERES). SEC12 is a type II ER transmembrane protein that functions as a guanine nucleotide exchange factor (GEF) for SAR1 while also recruiting SAR1 to the ER membrane (14, 15). GTP-bound SAR1 inserts its hydrophobic N-terminus into the ER membrane and recruits SEC23-SEC24 heterodimers to the ERES by directly interacting with SEC23 (16, 17). SEC23 stimulates SAR1 GTPase hydrolysis, thereby functioning as the GTPase-activating protein (GAP) for SAR1, while SEC24 mediates cargo recruitment and concentration in the ER lumen (18). Finally, SEC13 and SEC31 are recruited as an outer coat to complete the coat assembly (19) (Figure 1).

### Bulk flow and concentrative transport.

Bulk flow is the default secretory pathway in which proteins in the ER lumen can freely and passively leave the ER (20, 21). However, early immunoelectron microscopy and in vitro COPII reconstitution studies demonstrated that some secretory cargoes are selectively concentrated into COPII vesicles/tubules (22–25). Active cargo concentration implies interaction between COPII subunits on the cytoplasmic face of the ER membrane and secretory proteins within the ER lumen. While some transmembrane proteins can directly interact with SEC23/SEC24 in the COPII coat (26–28), other transmembrane and soluble cargoes (proteins without a transmembrane domain that are entirely constrained within the ER lumen) achieve physical interaction with the COPII coat via transmembrane cargo receptors (23) (Figure 1). Several transmembrane cargo receptors and adaptors have been identified in yeast and mammals, including LMAN1, SURF4, lectins such as VIP36, and p24 proteins (29), though it is unclear whether all or most secretory proteins require a cargo receptor for efficient secretion.

### Transport from the ER to the Golgi.

**Figure 2:**
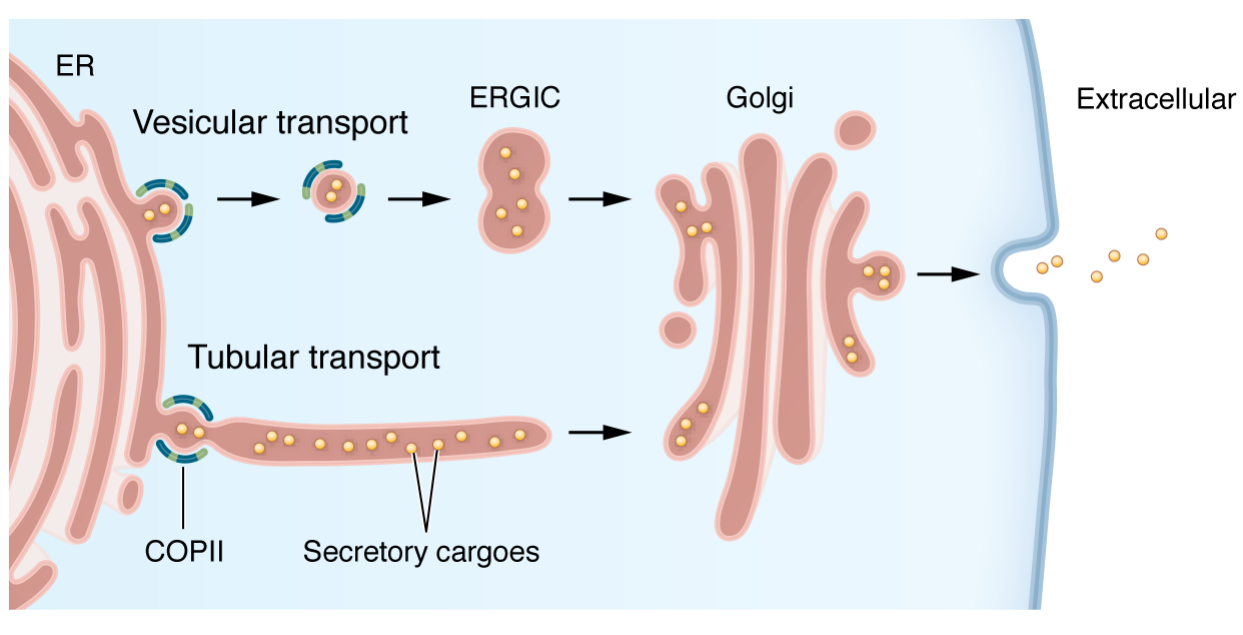
ER to Golgi transport of secreted proteins. Secretory proteins in the ER lumen are recruited into the COPII vesicle/tubule by COPII coat proteins. In the vesicular transport model, the vesicle buds from the ER and travels to the ERGIC/*cis*-Golgi network with COPII coat proteins accompanying the vesicle. In the tubular transport model, cargo proteins are transported in a continuous interwoven tubular network instead of discrete vesicles. COPII proteins remain on the ER membrane and function as a gatekeeper restricting entry of secretory proteins into tubules.

There are several models for how proteins are transported from one cellular compartment to another (30). From the early 1960s, two distinct models for the identity of transport carriers between the ER and the Golgi have been proposed: discrete vesicles (31) and continuous tubules/tunnels (32, 33). The foundation for the understanding of COPII-mediated ER to Golgi transport over the next few decades relied largely on the vesicular model, in which, following cargo recruitment, the outer coat (SEC13-SEC31) promotes ER membrane fission to generate discrete COPII vesicles 60–90 nm in size (34–37). These vesicles transport secretory proteins, along with the COPII coat proteins, to the ER-Golgi intermediate compartment (ERGIC) or the Golgi, where the vesicles fuse with the Golgi membrane, releasing their secretory cargo into the *cis*-Golgi network. The ERGIC is a stable membrane compartment between the rough ER and the Golgi that acts as the first post-ER sorting station for anterograde (to the Golgi) and retrograde (back to the ER) trafficking (38). Challenges to this model include the discovery of COPII-free transport carriers (39) and explaining the mechanism for transport of large cargoes such as procollagen (40) or prechylomicrons (41) whose diameters exceed the typical size of COPII vesicles. Recent studies using super-resolution imaging techniques on intact mammalian cells have visualized an interwoven ER network (42), suggesting that COPII coat proteins may remain on the ER membrane and function as a gatekeeper, restricting entry of secretory proteins into tubules rather than acting as an escort accompanying these proteins to the Golgi (43, 44) (Figure 2).

## Expansion of COPII paralogs in mammals

COPII proteins are highly conserved throughout eukaryotic evolution, and SEC23 and SEC24 orthologs have also been identified in Asgard, an archaea superphylum that is considered the closest prokaryotic relative of eukaryotes (45, 46). Comparative genomic studies identify at least one paralog for each of the five core COPII proteins (SAR1, SEC23, SEC24, SEC13, and SEC31) in every eukaryote genome analyzed, suggesting that all five are present in the last eukaryotic common ancestor (47, 48). Gene duplications have led to expansions of all five COPII proteins in multicellular plants and all but SEC13 in vertebrates (Table 1). Gene duplications are common evolutionary events. Typically, one duplicated copy accumulates loss-of-function mutations, becoming a pseudogene, and is eventually lost from the genome. Occasionally, both gene copies are conserved through neofunctionalization, in which one paralog acquires a new function, or subfunctionalization, in which the paralogs divide the functions of the ancestral gene. In the latter case, the division of functions can occur at the protein level or at the transcription level (49). A widely proposed explanation for the existence and conservation of multiple COPII paralogs in higher organisms is that each paralog has evolved unique functions to accommodate a more diverse secretome across different cell types. However, recent studies demonstrate that some paralogs have largely similar functions and can thus compensate for the loss of their partner paralog. Below, we will review and compare the reported human and mouse phenotypes associated with genetic deficiency for each COPII component as well as evidence for unique and/or redundant functions between paralogs.

## Specificity of COPII paralogs

### SAR1.

The first COPII protein recruited by SEC12 to the ERES is SAR1, which, in turn, recruits other COPII subunits to the ER membrane. Regulation of SAR1 GTPase kinetics by SEC23 and SEC31 is thought to be important for concentration of large cargo proteins (50). Most invertebrate genomes contain a single *SAR1* gene, whereas most vertebrates, including mammals, contain two paralogs: *SAR1A* and *SAR1B*. The human SAR1A and SAR1B paralogs differ at only 20 of 198 amino acid residues. Though the average degree of amino acid sequence identity for orthologous proteins between the human and mouse genomes is about 85%, each of the human and mouse SAR1 ortholog pairs exhibits about 98% sequence identity, differing in only three amino acids for SAR1A and two amino acids for SAR1B.

Despite this high degree of similarity between the two SAR1 paralogs, human genetic data suggest distinct functions for SAR1A and SAR1B. Although both *SAR1* paralogs are highly expressed in the intestine, only mutations in *SAR1B* have been linked to the rare autosomal recessive disorder chylomicron retention disease (CMRD, or Anderson’s disease) (51–53), and no human disorder has yet been associated with mutations in *SAR1A*. The approximately 1.5-fold increase in the expression of *SAR1A* in CMRD patients does not compensate for the loss of SAR1B (53). CMRD is characterized by failure to thrive and chronic diarrhea in infants due to malabsorption of dietary lipids and fat-soluble vitamins (54, 55). In CMRD patients, secretion of chylomicrons from the enterocytes is inhibited because of an inability to transport chylomicrons from the ER and/or abnormal fusion of prechylomicron transport vesicles to the Golgi — processes for which SAR1B appears to be required (56).

To explore the question of redundant/unique function between SAR1 paralogs at the molecular level, Melville et al. (57) identified three clusters of at least three amino acid sequence differences between SAR1A and SAR1B. Two of these clusters are highly conserved among mammals, reptiles, and birds. One cluster is adjacent to the GTP-binding pocket, and the other is on an α-helix near the known binding site of SEC31 on SEC23, which might influence SAR1 interaction with the SEC23-SEC31 complex. Further analyses demonstrated a faster GTPase exchange rate for SAR1A than for SAR1B, with a swap of the amino acid cluster adjacent to the GTP-binding pocket reversing this difference between the two paralogs. In contrast, SAR1B binds more strongly to SEC23 than does SAR1A, and swapping the α-helix amino acid cluster reverses SEC23 affinity. These data indicate that SAR1A and SAR1B have some distinct biochemical properties at the molecular level.

However, limited in vitro data suggest some degree of functional overlap for these SAR1 paralogs. Inactivation of *SAR1B* in human Caco-2/15 cells, a chylomicron-secreting cell line, results in decreased chylomicron secretion, disrupted lipid homeostasis, and increased oxidative stress (58, 59). SAR1A-null Caco-2/15 cells exhibit a similar but less dramatic phenotype (59). Combined deletion of SAR1A and SAR1B was required to recapitulate the more severe phenotype observed in patients with CMRD. This discrepancy between in vivo and in vitro could potentially be explained by relative expression levels of *SAR1A* and *SAR1B*. *SAR1B* is expressed at approximately 3-fold higher levels than *SAR1A* in human intestine (53), while *SAR1A* is expressed at slightly higher levels than *SAR1B* in Caco-2/15 cells, with a further approximately 1.3-fold increase in *SAR1A* expression following *SAR1B* deletion (58). In mice, loss of expression from a single *Sar1b* allele is sufficient to recapitulate the reduced chylomicron secretion phenotype seen in humans, with homozygous *Sar1b* deletion in mice resulting in late-gestation lethality (60). Taken together, these data suggest at least some degree of functional overlap between SAR1A and SAR1B, with the total level of SAR1 expression providing an important determinant for CMRD manifestations.

### SEC23.

SEC23 is a cytosolic protein that forms a heterodimer with SEC24. The SEC23-SEC24 complex is recruited to the ER membrane by SAR1 and, together with SAR1, forms the inner COPII coat, which recruits cargo proteins from the ER lumen (61, 62). Though invertebrate genomes generally encode a single *SEC23* gene, most vertebrate genomes, including mammal genomes, encode two *SEC23* paralogs, *SEC23A* and *SEC23B* (48). The *SEC23* gene duplication is estimated to have occurred about 615 million years ago. The two mammalian *SEC23* paralogs share about 85% sequence identity at the amino acid level, whereas the human and mouse SEC23A and SEC23B orthologs are about 98% and about 97% identical at the protein level, respectively (63).

Human genetic data again suggest that the *SEC23* paralogs have evolved divergent functions. Despite ubiquitous expression of both *SEC23A* and *SEC23B*, loss-of-function mutations in each paralog lead to different disorders affecting distinct cell types. Mutations in human *SEC23A* result in defective collagen secretion leading to the autosomal recessive condition cranio-lenticulo-sutural dysplasia (CLSD), characterized by abnormal cranial fontanel closures, facial dysmorphisms, skeletal abnormalities, and sutural cataracts (64, 65). In contrast, mutations in *SEC23B* result in congenital dyserythropoietic anemia type II (CDAII), an autosomal recessive disorder characterized by anemia and increased numbers of bi/multinucleated red blood cell precursors in the bone marrow (66, 67). Additionally, expression of human SEC23A and not SEC23B has been shown to rescue Sec23p-null yeast, which is also consistent with unique functions for the two mammalian SEC23 paralogs (68).

Initial results from mouse models also suggested distinct, though potentially partially overlapping, functions for SEC23A and SEC23B. *Sec23a-*null mice exhibit mid-embryonic lethality associated with defective extraembryonic membrane development and neural tube closure in the midbrain, likely due to impaired secretion of multiple collagen types (69), and consistent with the collagen secretion defect observed in humans with CLSD. However, in contrast to human SEC23B deficiency, SEC23B-deficient mice exhibit an entirely normal red blood cell phenotype (70, 71), instead demonstrating perinatal lethality due to degeneration of the pancreas (70, 72). There is also no evidence for altered collagen secretion in *Sec23b*-null mice (70). Notably, complete loss of one SEC23 paralog in combination with haploinsufficiency of the remaining paralog results in embryonic death at an earlier developmental time (69), suggesting some degree of overlapping functions between the paralogs.

Though it was previously demonstrated that only human SEC23A can rescue Sec23p deficiency in yeast, a more recent study shows that murine and human SEC23A and SEC23B are each individually sufficient to complement the loss of Sec23p in yeast, and delivery of a *sec23a* transgene rescues the lethality of Sec23b deficiency in zebrafish (63). Additionally, substitution of the SEC23A coding sequence for that of SEC23B at the endogenous *Sec23b* locus fully rescued the perinatal lethal pancreatic degeneration seen in SEC23B*-*deficient mice, with no apparent abnormalities in these adult animals expressing only SEC23A sequences, but under the regulatory control of both the endogenous *Sec23a* and *Sec23b* genes (63). Similarly, SEC23A has been demonstrated to overlap in functions with SEC23B in human erythroid cells, with increased SEC23A expression being sufficient to rescue the erythroid differentiation defect in SEC23B-deficient cells (73). Taken together, these data demonstrate that the two mammalian SEC23 paralogs exhibit highly overlapping and potentially identical functions, with the discordant phenotypes observed between SEC23A and SEC23B deficiencies, and between humans and mice, resulting primarily from evolutionary differences in tissue-specific gene expression programs. Consistent with this hypothesis, murine *Sec23a* gene expression has been shown to be maintained throughout erythropoiesis, in contrast to human *SEC23A* expression, which declines rapidly upon induction of terminal erythroid differentiation (74), potentially explaining the absence of a red blood cell phenotype in mice with hematopoietic deficiency of SEC23B (71). Indeed, inactivation of all four *Sec23* alleles in erythroid cells is required to reproduce the CDAII phenotype in mice (73). While subtle differences in biochemical properties between the SEC23 paralogs (as between the SAR1 paralogs) cannot be excluded, these data suggest that mammalian SEC23A and SEC23B are largely functionally interchangeable.

### SEC24.

SEC24 is the primary COPII component responsible for cargo selection via either direct interaction with an ER exit signal on the cytoplasmic domain of the cargo protein itself (in the case of transmembrane proteins) or an indirect interaction mediated through a cargo receptor (for soluble cargoes restricted to the ER lumen) (26). SEC24 has also been implicated in autophagy of the ER (ER-phagy), with SEC24A/B involved in bulk ER-phagy (75), whereas SEC24C is required for selective ER-phagy (76). We refer readers to other reviews for a more detailed discussion of ER-phagy and crosstalk between the secretory and autophagy pathways (77, 78).

Several cargo binding sites on SEC24 have been mapped (27). SEC24 is the only COPII component encoded by more than one paralogous gene in yeast (*Sec24*, *Lst1*, and *Iss1*). Yeast Sec24p shares 55% and 23% protein sequence identity with Iss1p and Lst1p, respectively, with overexpression of Iss1p, but not Lst1p, sufficient to rescue Sec24p deficiency (79–81). Mammalian genomes encode four SEC24 paralogs, SEC24A–SEC24D, with the SEC24A and SEC24B subgroup more similar to yeast Sec24p and Iss1p, and the SEC24C and SEC24D subgroup more similar to yeast Lst1p (82, 83). Analysis of available eukaryotic SEC24 sequences suggests the presence of at least three SEC24 paralogs in the last common eukaryotic ancestor (48). Human *SEC24* paralogs share about 50% sequence identity within and about 25% sequence identity across subgroups at the amino acid level. All SEC24 paralogs contain a highly conserved C-terminal region and a hypervariable N-terminal segment (84). Given the role of SEC24 in cargo recruitment, the expansion of COPII paralogs is thought to have been driven to accommodate a greater diversity of secretory cargoes in mammals. Current evidence suggests that SEC24 paralogs within the same subgroup (SEC24A/B versus SEC24C/D) may exhibit largely but not entirely overlapping function, with larger differences in cargo-sorting signal affinity between the two subgroups (85–88).

No human disorders have been associated with mutations in *SEC24A* or *SEC24C*. Compound heterozygosity for loss-of-function mutations in *SEC24D* has been reported to result in a syndromic form of osteogenesis imperfecta (89). Though heterozygous *SEC24B* missense variants were reported in 4 of 163 cases of neural tube defects (NTDs) in one study (90), these and other missense variants are present in unaffected individuals in the Genome Aggregation Database (gnomAD) (91), arguing against a significant association of heterozygous *SEC24B* mutations with NTDs. In contrast, a wide range of phenotypes have been reported in mice genetically engineered to be deficient in each of the four SEC24 paralogs. SEC24A-deficient mice demonstrate normal development and survival with the only identifiable phenotype being moderate hypocholesterolemia due to impaired secretion of PCSK9, a plasma protein that negatively regulates low-density lipoprotein (LDL) receptor abundance and, thereby, LDL clearance from circulation (92). SEC24B-deficient mice exhibit late embryonic lethality at approximately E18.5 due to a neural tube closure defect, likely resulting from decreased trafficking of VANGL2, a planar-cell polarity protein (93). Notably, no NTD phenotype was observed in heterozygous *Sec24b^+/–^* mice, further arguing against an association between heterozygous mutations in human *SEC24B* and NTDs. Ubiquitous loss of murine SEC24C results in early embryonic lethality at approximately E7.5 due to abnormal gastrulation and ectoderm development (94), while mice with SEC24C deficiency restricted to neural progenitors demonstrate perinatal lethality and microcephaly due to widespread cell death (95). Lastly, absence of SEC24D results in early embryonic death at or before the eight-cell stage (96). A recent proteomic study using an in vitro vesicle reconstitution system demonstrated that SEC24C and SEC24D preferentially interact with ERGIC1 whereas SEC24A favors CNIH4 (97), a proposed cargo receptor for G protein–coupled receptors (98). Several other cargo-specific preferences for each SEC24 paralog have also been reported, including preference of the cargo PCSK9 for SEC24A/B (92); of VANGL2 for SEC24B (93); of SERT (99), SLC6A14 (100), and autotaxin (101) for SEC24C; and of GABA transporter 1 (GAT1) (102) for SEC24D. The glycine transporter (GLYT1) has also been demonstrated to physically interact with SEC24D, though it is unclear whether this interaction is exclusive to SEC24D, as interactions with the other SEC24 paralogs were not tested in this study (103).

In contrast to this evidence for cargo specificity, other studies suggest significant overlap in cargo repertoire for SEC24 paralogs, especially between those within the same subgroup. The vesicular stomatitis virus G glycoprotein (VSV-G) has been reported to interact strongly with mammalian SEC24A/B but not SEC24C/D, whereas syntaxin 5 and membrin are specifically packaged by mammalian SEC24C/D (84). Similarly, in *Sec24a*-null mice, inactivation of an additional *Sec24b* allele results in a further approximately 25% reduction in plasma cholesterol, consistent with partial overlap in function between murine SEC24A and SEC24B (92). In contrast, the human recycling transmembrane protein p24-p23, which acts as a cargo receptor for GPI-anchored CD59, prefers SEC24C/D for ER export (104). Lastly, replacement of the majority of the *Sec24c* coding sequence with *Sec24d* at the endogenous *Sec24c* locus partially rescues the embryonic lethal *Sec24c*-null phenotype, again suggesting significant functional overlap between SEC24C and SEC24D (87). Taken together, these data demonstrate partial functional overlap between SEC24 paralogs within the same subgroup and divergence between the two subgroups.

### SEC31.

Heterotetramers of SEC31 and SEC13 form the outer COPII coat (105–107). The SEC13-SEC31 complex mediates membrane deformation. SEC31 also directly interacts with SEC23 to stimulate its GTPase-activating protein (GAP) activity, thereby triggering SAR1 GTP hydrolysis (108). It has been suggested that SEC31 fine-tunes SEC23 GAP kinetics to accommodate large cargoes such as collagen (109, 110). Indeed, downregulation of SEC31 as a result of SEC13 depletion in HeLa cells leads to impaired secretion of collagen but not of the temperature-sensitive VSV-G–ts045 glycoprotein (111). VSV-G is a viral protein that is evolutionarily optimized for ER-Golgi export, and experimentation with this protein requires overexpression and culturing of cells at high temperature for a prolonged period. It is unclear whether deletion of SEC31 would produce the same effect for physiologic cargoes. Mammalian genomes encode two paralogs of SEC31 (*SEC31A* and *SEC31B*), while yeast contains only a single *Sec31* gene. *SEC31A* is highly expressed in most human tissues, except for the brain, whereas *SEC31B* is expressed at low levels in most tissues, except for the cerebellum and testis. Human SEC31A shares about 45% sequence identity with SEC31B, and these two paralogs share about 26% and about 19% sequence similarity, respectively, with yeast Sec31p at the amino acid level (112–114). Human *SEC31B* also appears to be alternatively spliced, producing a C-terminally truncated protein that is about 41% of the full-length SEC31B, though the function of this truncated SEC31B is unclear (114). No human disorder has been associated with mutations in either *SEC31* paralog, and mouse models have also not yet been reported.

Though there are currently no published data to assess potential functional overlap among vertebrate SEC31 paralogs, there is some evidence for such overlap in plants. The *Arabidopsis* genome also encodes two *SEC31* paralogs, which share about 59% protein sequence identity with each other and about 25% protein sequence identity with their human SEC31 orthologs. *SEC31B*-mutant *Arabidopsis* are infertile because of a defect in pollen development (115), whereas *SEC31A*-deficient *Arabidopsis* exhibit normal vegetative and reproductive development. Inactivation of both *SEC31A* and *SEC31B* results in lethality due to impaired gametogenesis (116). *SEC31B* is expressed at about 600-fold higher levels in most plant tissues than *SEC31A*. However, *SEC31B-*null transgenic plants in which SEC31A expression is driven by the *SEC31B* promoter exhibit normal fertility (116), demonstrating significant functional overlap between these two plant paralogs, with their evolutionary conservation likely driven by their divergent gene expression programs.

### SEC13.

SEC13, together with SEC31, forms the outer COPII coat complex. SEC13 also interacts with several proteins of the nuclear pore complex and shuttles between the nucleus and the cytoplasm (117–119). The single human *SEC13* gene shares about 97% and about 46% sequence identity with mouse and yeast SEC13, respectively. Though *SEC13* mutations are not associated with any known human disorder, mice with complete loss of SEC13 are not viable. However, mice with low levels of residual SEC13 appear grossly normal, though exhibiting aberrant expression of several genes involved in immune response and inflammation (120). In zebrafish, deletion of *sec13* leads to impaired retinal and gut development associated with a procollagen secretion defect (121, 122). Consistent with these observations, depletion of SEC13 in human intestinal epithelial (Caco-2) cells results in aberrant cyst morphogenesis (123).

## Specificity of cargo receptors

As previously noted, cargo receptors are ER transmembrane proteins that bridge the interaction between cargoes in the ER lumen and COPII proteins on the cytoplasmic face of the ER. Several cargo receptors have been described in mammals, including LMAN1 (ERGIC53) and SURF4 (124, 125). The following discussion will focus on LMAN1 and SURF4, which have been extensively studied in vitro and in vivo. For a more comprehensive discussion of other putative ER cargo receptors, the reader is referred to ref. 29.

### LMAN1.

LMAN1 (also known as ERGIC53) is a 53 kDa type I transmembrane protein that was originally identified as a marker for the ERGIC (126). LMAN1 resides primarily in the ER/ERGIC lumen, with a short C-terminal tail of 12 amino acids, including a dilysine diphenylalanine motif (KKFF), extending into the cytoplasm. The FF motif is required for ER export whereas the KK motif is necessary for ER retrieval (127, 128), facilitating LMAN1 cycling between the ER, ERGIC, and *cis*-Golgi. The LMAN1 luminal segment includes an L-type lectin domain that binds to mannose in a Ca^2+^-dependent manner (129, 130). LMAN1 was initially suspected to function as a cargo receptor, recruiting secretory proteins in the ER lumen and escorting them to the Golgi, based on its homology to the well-characterized yeast cargo receptor Emp47p (131). The identification of loss-of-function mutation in *LMAN1* as the cause of the autosomal recessive human bleeding disorder combined factor V and factor VIII deficiency (F5F8D) (132, 133) identified these two proteins as putative cargoes for LMAN1. F5F8D patients exhibit plasma levels of coagulation factor V (FV) and factor VIII (FVIII) reduced to 5%–30% of normal (134). Further studies identified other potential LMAN1 cargoes, including the lysosomal proteins cathepsin C (CTSC) (135) and cathepsin Z (CTSZ) (136), membrane protein GABA type A receptors (GABAARs) (137), and other secreted proteins including α_1_-antitrypsin (A1AT) (138), Mac-2–binding protein (Mac-2BP) (139), and matrix metalloproteinase-9 (MMP-9) (140).

Mutations in *LMAN1* account for about 70% of cases of F5F8D (141), with the remaining ~30% of cases due to inactivating mutations in *MCFD2* (142). Together, mutations in *LMAN1* and *MCFD2* appear to account for all cases of F5F8D (143). MCFD2 is a 16 kDa soluble EF-hand–containing protein that lacks an ER retrieval motif and is retained in the ER by forming a stable, Ca^2+^-dependent 1:1 stoichiometry complex with LMAN1 (142, 144). In the absence of LMAN1, MCFD2 is efficiently secreted (145). For efficient ER exit, dimerization of LMAN1 is required (146); however, LMAN1 hexamers have also been observed (147, 148).

The recognition motif for LMAN1/MCDF2-dependent cargoes is unclear, and the LMAN1 cargo repertoire appears to be limited (149). The few known LMAN1/MCFD2 cargoes have been identified by various approaches, including overexpression of an ER-trapped LMAN1 mutant in HeLa cells (CTSC; ref. 135), fluorescent-based protein fragment complementation assays (CTSZ, A1AT, Mac-2BP, and MMP-9; refs. 136, 138–140), and mass spectrometry following coimmunoprecipitation (GABAARs; ref. 137). Most of these putative cargoes are heavily glycosylated, with LMAN1 binding shown to be carbohydrate dependent for CTSC, CTSZ, A1AT, Mac-2BP, and MMP-9 (124, 135, 138–140), though not required for FVIII or GABAARs (137, 144). In the absence of LMAN1, MCFD2 can still bind to FVIII; however, it is unclear whether LMAN1 can independently interact with FVIII in the absence of MCFD2 (144). MCFD2 is required for efficient secretion of FV, FVIII, A1AT, and Mac-2BP (139, 144, 150) but is dispensable for CTSC and CTSZ (145). Dependence of FV, FVIII, and A1AT on LMAN1 has been confirmed in vivo, with *Lman1*-deficient mice exhibiting reduced plasma levels of FV and FVIII and ER accumulation of A1AT in hepatocytes. However, similar accumulation of CTSC and CTSZ was not observed (151).

### SURF4.

SURF4 is a 29 kDa protein with five transmembrane domains localizing to the ER and ERGIC (152). Similarly to LMAN1, SURF4 also displays a dilysine ER retrieval motif at its cytoplasm-facing C-terminus, facilitating its retention in the ER (153). Erv29p, the SURF4 ortholog in yeast, is the well-characterized cargo receptor for pro–α-factor, carboxypeptidase Y, and proteinase A (154–156). SURF4 and Erv29p are highly conserved across eukaryotes, with orthologs also identified in *Caenorhabditis* *elegans* and *Drosophila* (157). While Erv29p is dispensable in yeast, SFT-4 (the *C*. *elegans* ortholog of *SURF4*) and SURF4 are required for survival in *C*. *elegans* and mice, respectively (158, 159). SURF4 was shown to function in conjunction with LMAN1 to maintain ERGIC and Golgi structural integrity (152) and, based on its cellular localization and sequence homology to Erv29p, was long suspected to function as a cargo receptor in mammalian cells, though putative cargoes have only recently been identified.

No human disorder has been associated with mutations in *SURF4*. However, genetic polymorphism at the *SURF4* gene is strongly associated with reduced plasma lipid levels and reduced cardiovascular risk in human populations (160). As noted above, efficient secretion of PCSK9 is specifically dependent on SEC24A, with their localization to opposite sides of the ER membrane implying a requirement for a specific cargo receptor serving as the physical link (92). A whole-genome CRISPR screen in human embryonic kidney cells (HEK293T) identified SURF4 as this putative PCSK9 cargo receptor (125). A recent report confirmed PCSK9 secretion dependence on both SEC24A and SURF4 in HEK293T and human hepatic (HuH7) cells. In addition, the authors demonstrated that disruption of SEC24A-SURF4 binding by a small molecule, 4-phenylbutyrate, inhibits PCSK9 secretion, further supporting the role for SURF4 as a cargo receptor that bridges PCSK9 and SEC24A (161). SFT-4 was also shown to be essential for the trafficking of the yolk protein VIT-2, a component of *C*. *elegans* lipoproteins that share about 22% sequence identity with human apolipoprotein B (APOB); this same group demonstrated that SURF4 also serves as a cargo receptor for APOB in human hepatic HepG2 cells (162). Several other SURF4 cargoes have been identified, including growth hormone, dentin sialophosphoprotein (DSPP), amelogenin (163), erythropoietin (164), pathogenic A1AT polymers (165), sonic hedgehog (166), proinsulin (167), and the lysosomal proteins progranulin and prosaposin (168).

Further analysis of multiple potential SURF4 cargoes across multiple vertebrate species led to the proposal of a highly conserved hydrophobic-proline-hydrophobic tripeptide (ER-ESCAPE) motif downstream of the signal peptide for many putative SURF4 cargoes (163). Deletion of *SURF4* in human cells significantly reduced the secretion of some proteins with this ER-ESCAPE motif (163). However, several putative SURF4 cargoes noted above do not carry this ER-ESCAPE motif, suggesting the presence of other recognition motifs that have not yet been identified. Proteomic analysis of conditioned media collected from SURF4-deficient cells revealed several SURF4 clients in HEK293T and HuH7 cells, half of which carry the ER-ESCAPE motif or a Cardin-Weintraub motif, which has also been shown to mediate interaction with SURF4 (161, 166). Interestingly, while replacement of the ER-ESCAPE tripeptide motif with glutamic acids (EEE) significantly impairs secretion of several putative SURF4 cargoes (161, 163), APOB, arguably the most well-studied SURF4 cargo in both in vitro and in vivo models, carries the EEE motif downstream of the signal peptide. Taken together, these data suggest a more complex process governing cargo recognition by SURF4.

The above reports suggest that SURF4 may play a role in the trafficking of a much broader range of secretory cargoes compared with LMAN1. Proteomic analysis of in vitro–reconstituted COPII vesicles are also consistent with this model, with SURF4 deletion resulting in the depletion of many more proteins from reconstituted COPII vesicles compared with LMAN1 deletion (149). A recent study demonstrated that SURF4 traffics ER cargoes into an elongated tubular ERGIC that lacks LMAN1. This tubular ERGIC accelerates ER to Golgi trafficking of SURF4 cargoes, suggesting a distinct SURF4 trafficking route (169).

## Concluding remarks

Although the conventional ER-Golgi secretory pathway was first identified over five decades ago, a number of key questions remain unanswered. Are the physical carriers that transport secreted proteins from the ER to the Golgi discrete vesicles, elongated tubules, or continuous tunnels that physically connect the two compartments? Early studies by Schekman and colleagues in yeast established the widely accepted COPII vesicle model. However, recent advances in super-resolution live-cell imaging demonstrate the presence of elongated tubules originating from the ERES, with the COPII proteins appearing to function as gatekeepers at the ERES collar rather than as escorts that travel with cargoes to the Golgi. While superficially conflicting, these models are not mutually exclusive, and multiple different pathways may exist, with the path that each specific protein takes depending on its size, relative abundance in the ER lumen, and requirement for a cargo receptor to facilitate its secretion.

Next, although a diverse set of secretory cargoes are transported from ER to Golgi, it remains unclear how many require a cargo receptor to facilitate this process. The thousands of known secreted proteins and the very limited number of putative cargo receptors identified to date are suggestive of a role for a passive bulk flow mechanism, with only a small subset of secreted proteins requiring interaction with a cargo receptor. Alternatively, multiple additional cargo receptors may remain to be identified. Furthermore, even for the small subset of proteins clearly demonstrated to be dependent on a specific cargo receptor for secretion, deletion of the corresponding cargo receptor does not result in complete blockade of secretion, suggesting the presence of an alternative “backup” cargo receptor(s) or another mechanism for basal protein transport, with the cargo receptor only required for maximal secretion efficiency.

Finally, a better understanding of the functional conservation of COPII paralogs might have translational relevance for human diseases resulting from deficiency of a specific COPII protein paralog, such as CMRD (SAR1B) or CDAII (SEC23B). For COPII paralogs exhibiting extensive or complete functional overlap, therapies that upregulate expression of one paralog could potentially compensate for genetic deficiency of the other (73). In addition, future insights into cargo–cargo receptor interactions and binding motifs could facilitate fine-tuning of secretion for a specific cargo protein. Lastly, optimization of cargo–cargo receptor interactions could potentially be leveraged to improve efficiency of recombinant protein production.

## Figures and Tables

**Table 1:**
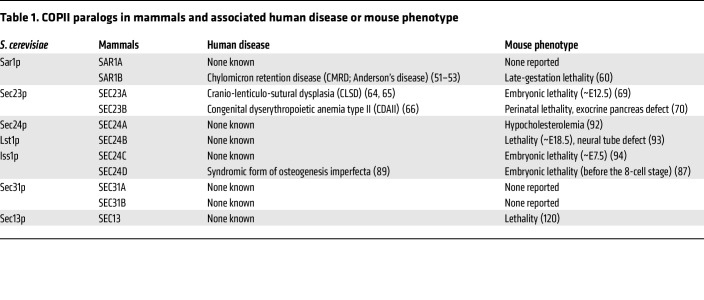
COPII paralogs in mammals and associated human disease or mouse phenotype

## Version 1 01/03/2023

Electronic publication
